# Hemichannel-mediated release of lactate

**DOI:** 10.1177/0271678X15611912

**Published:** 2015-10-23

**Authors:** Anastassios Karagiannis, Sergiy Sylantyev, Anna Hadjihambi, Patrick S Hosford, Sergey Kasparov, Alexander V Gourine

**Affiliations:** 1 Department of Neuroscience, Physiology and Pharmacology, Centre for Cardiovascular and Metabolic Neuroscience, University College London (UCL), London, UK; 2 Centre for Clinical Brain Sciences, University of Edinburgh, Edinburgh, UK; 3 Department of Physiology and Pharmacology, University of Bristol, Bristol, UK

**Keywords:** Astrocytes, connexin, lactate, metabolism, monocarboxylate transporters, pannexin

## Abstract

In the central nervous system lactate contributes to the extracellular pool of readily available energy substrates and may also function as a signaling molecule which mediates communication between glial cells and neurons. Monocarboxylate transporters are believed to provide the main pathway for lactate transport across the membranes. Here we tested the hypothesis that lactate could also be released via opening of pannexin and/or functional connexin hemichannels. In acute slices prepared from the brainstem, hippocampus, hypothalamus and cortex of adult rats, enzymatic amperometric biosensors detected significant tonic lactate release inhibited by compounds, which block pannexin/connexin hemichannels and facilitated by lowering extracellular [Ca^2+^] or increased *P*CO_2_. Enhanced lactate release triggered by hypoxia was reduced by ∼50% by either connexin or monocarboxylate transporter blockers. Stimulation of Schaffer collateral fibers triggered lactate release in CA1 area of the hippocampus, which was facilitated in conditions of low extracellular [Ca^2+^], markedly reduced by blockade of connexin hemichannels and abolished by lactate dehydrogenase inhibitor oxamate. These results indicate that lactate transport across the membranes may occur via mechanisms other than monocarboxylate transporters. In the central nervous system, hemichannels may function as a conduit of lactate release, and this mechanism is recruited during hypoxia and periods of enhanced neuronal activity.

## Introduction

Neurons require constant and sufficient supply of energy substrates to meet their metabolic needs. Lactate is one of the key elements of brain energy metabolism and contributes to the extracellular pool of readily available energy substrates. The so-called ‘astrocyte-to-neuron lactate shuttle’ hypothesis suggests that neuronal activity is fuelled by lactate provided by neighbouring astrocytes.^1,2^ Although there is evidence which argues against the importance (and existence) of astrocyte-to-neuron lactate shuttle (for a comprehensive review see Dienel^3^), the role of lactate and its effective transport in supporting metabolic needs of the brain may become highly significant in certain conditions. For example, physical activity and exercise are associated with progressive increase in brain lactate uptake (from the blood) and oxidation as work load and plasma lactate levels rise.^4,5^ In addition to its metabolic role, lactate may also function as a signaling molecule which in the brain mediates communication between astrocytes and neurons.^6–9^

In order to fulfil metabolic (at rest or during exercise) and/or signaling roles, lactate requires an effective mechanism of transport across the membrane(s) – the process which is currently attributed to the operation of monocarboxylate transporters (MCTs).^10^ This, however, does not exclude the involvement of other potential mechanisms.^11^ Gap junction hemichannels have been reported to be permeable to various molecules including adenosine 5'-triphosphate (ATP), glutamate and glucose,^12–18^ and in this study we tested the hypothesis that lactate could be released into the extracellular space via opening of pannexin and/or functional (i.e. unopposed) gap junction connexin hemichannels.

## Materials and methods

All the experiments were performed in accordance with the European Commission Directive 2010/63/EU (European Convention for the Protection of Vertebrate Animals used for Experimental and Other Scientific Purposes) and the UK Home Office (Scientific Procedures) Act (1986) with project approval from the UCL Institutional Animal Care and Use Committee.

### *In vitro* slice preparations

Young adult Sprague-Dawley rats (∼120 g) were terminally anesthetized with halothane inhalation overdose, the brains were removed and placed in ice cold artificial cerebrospinal fluid (aCSF) containing 124 mM NaCl, 26 mM NaHCO_3_, 3 mM KCl, 2 mM CaCl_2_, 1.25 mM NaH_2_PO_4_, 1 mM MgSO_4_, 10 mM Glucose saturated with 95% O_2_/5% CO_2_ (pH 7.4) with an addition of 9 mM Mg^2+^. Horizontal brainstem slices containing the intact ventral surface (thickness ∼400 µm) or coronal brainstem, hypothalamic or cortical slices (thickness 300 µm) were cut using a vibratome and then incubated at room temperature for 1 h in a standard aCSF saturated with 95% O_2_/5% CO_2_.

### Biosensor recordings

**Figure 1.**
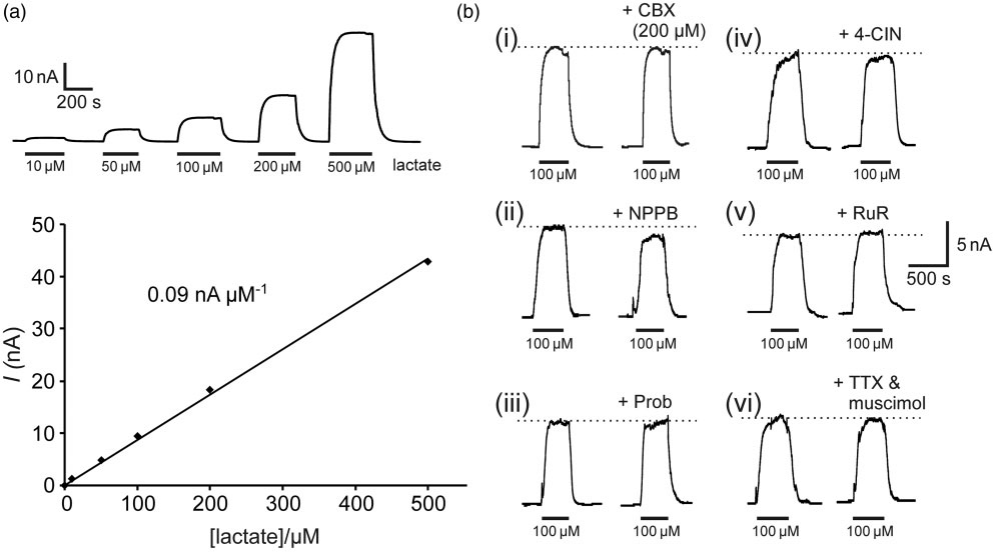
Performance of the lactate biosensors. (a) Current (*I*) calibration curve and sample responses of the lactate biosensor demonstrating linearity of analyte detection in concentrations from 10 μM to 500 μM; (b) Insensitivity of the lactate biosensor detection system to pharmacological agents used in this study. Traces illustrate sample biosensor current responses to lactate (100 µM) when calibrated in control artificial cerebrospinal fluid (aCSF), and then in the presence of (i) carbenoxolone (CBX), (ii) 5-nitro-2 -(3-phenylpropylamino)-benzoic acid (NPPB, 200 µM), (iii) probenecid (Prob, 150 µM), (iv) α-cyano-4-hydroxycinnamate (4-CIN, 250 µM), (v) Ruthenium red (RuR, 20 µM), and (vi) tetrodotoxin (TTX, 1 µM) + muscimol (100 µM).

**Figure 2.**
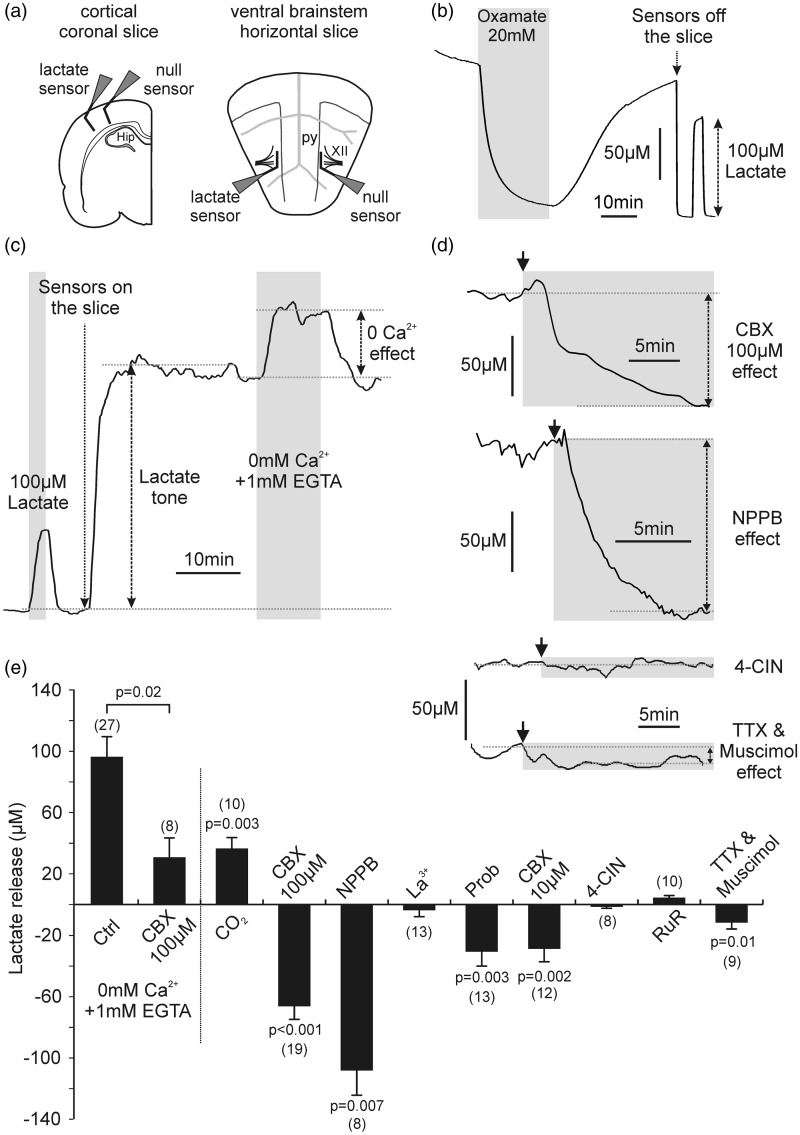
Hemichannel-mediated tonic release of lactate. (a) Schematic illustration of a dual recording configuration of lactate and null (control) biosensors placed in a direct contact with the surface of the coronal cortical slice or uncut ventral surface of the horizontal brainstem slice. Difference in current between lactate and null biosensors was used to determine the amount of lactate release. XII hypoglossal rootlets; (b) Representative example of oxamate-induced changes in lactate release detected by the biosensors placed on the surface of the brainstem slice followed by calibration with 100 µM lactate; (c) Representative example of changes in lactate biosensor current during calibration by application of 100 µM lactate, after biosensor placement in a direct contact with the surface of the brainstem slice (revealing lactate tone), and in response to lowering extracellular [Ca^2+^] (indicated by grey shading); (d) Representative recordings of lactate biosensor current showing changes in lactate tone in response to application of CBX (100 µM), NPPB (200 µM), 4-CIN (250 µM), or co-application of TTX (1 µM) and muscimol (100 µM). Period of drug application is indicated by grey shading; (e) Summary data illustrating peak changes in tonic lactate release in response to lowering extracellular [Ca^2+^] (in the absence and presence of CBX, 100 µM), increases in *P*CO_2_, or application of compounds known to inhibit functional connexin hemichannels (CBX, 100 µM; NPPB, 200 µM; La^3+^, 100 µM), pannexin hemichannels (CBX, 10 µM; Prob, 150 µM), monocarboxylate transporters (4-CIN, 250 µM), CALHM1 channels (RuR, 20 µM) or suppress the neuronal activity (TTX, 1 µM + muscimol, 100 µM). Mean ± SEM. *p* values – Wilcoxon signed-rank test. Hip: hippocampus; py: pyramidal tract.

Release of lactate was recoded in real-time using amperometric enzymatic lactate biosensors (Sarissa Biomedical) placed in a direct contact with the surface of the slice. The operation of the lactate biosensor is based on enzymatic activity of lactate oxidase which, in the presence of oxygen, converts lactate to pyruvate and H_2_O_2_. The latter is detected electrochemically. Figure 1 illustrates linearity of lactate detection by the biosensors in concentrations from 10 μM to 500 μM and insensitivity of the lactate biosensor detection system to pharmacological agents used in the current study. A dual recording configuration of a null sensor (lacking lactate oxidase) and lactate biosensor was used (Figure 2(a)), as described previously.^19–22^ The null sensor served as a control to determine whether any non-specific electroactive interferents were released and confound the measurements. Null sensor currents were subtracted from the lactate biosensor currents. Biosensors were calibrated with known amount of lactate (10 or 100 µM) directly in the slice chamber (i.e. in the identical temperature, aCSF composition/osmolarity conditions) immediately before and after the recordings (Figure 2(b) and (c)). To convert changes in sensor current to changes in lactate concentration, the means of the initial and final calibrations were used.

**Figure 3.**
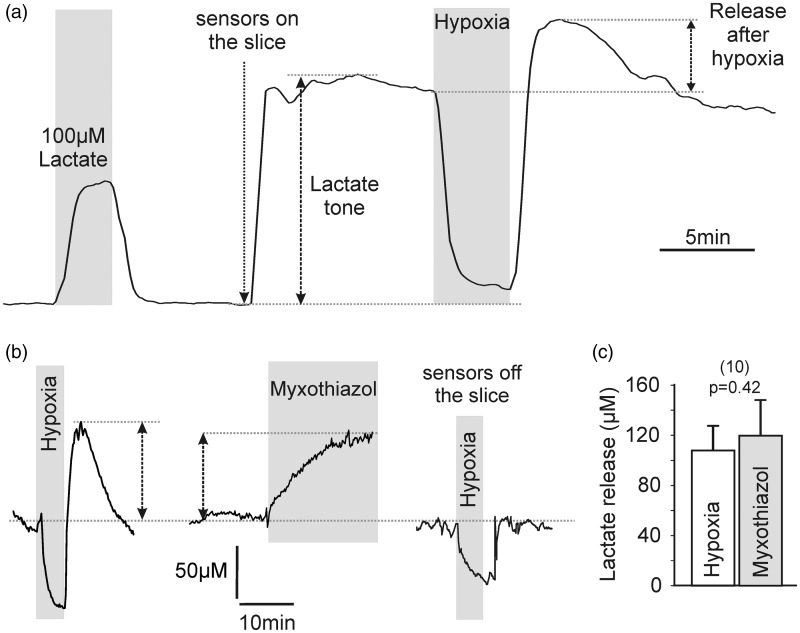
Facilitated release of lactate in response to hypoxia. (a) Representative example of changes in lactate biosensor current during calibration, after biosensor placement on the surface of the brainstem slice, and in response to a hypoxic challenge (perfusion with aCSF saturated with 95% N_2_/5% CO_2_). Peak hypoxia-induced lactate release is measured upon re-oxygenation. (b) Representative examples of changes in lactate biosensor current recorded during and after hypoxia when the sensor is placed on the surface of the brain slice (left) or in the bath away from the slice (right) and on the surface of the slice during application of myxothiazol (middle). Decrease in O_2_ availability reduces biosensor current followed by a positive signal upon re-oxygenation (hypoxia-induced lactate release), which is only observed when the sensor is in a direct contact with the brain tissue. (c) Peak lactate efflux of a similar magnitude is triggered by hypoxia and myxothiazol.

Recordings were made from the slice placed on an elevated grid in a flow chamber at 35℃. Isohydric hypercapnia was mimicked by replacing normal aCSF with a solution containing 70 mM NaCl, 80 mM NaHCO_3_, 3 mM KCl, 2 mM CaCl_2_, 1.25 mM NaH_2_PO_4_, 1 mM MgSO_4_, 10 mM Glucose saturated with 12% CO_2_ (*P*CO_2_ 70–80 mmHg, pH 7.4). Hypoxic conditions were induced for 2–4 min by replacement of oxygen in the medium with nitrogen (perfusion of the chamber with aCSF saturated with 95% N_2_/5% CO_2_). The effect of hypoxia was determined by measuring the peak lactate release upon re-oxygenation (Figure 3(a)) as previously described.^22^ This represents an accurate estimation of peak hypoxia-induced lactate release since similar release is triggered by application of a mitochondrial complex III inhibitor myxothiazol (2.5 µM) (Figure 3(b) and (c)).

### Electrophysiology

Coronal hippocampal slices (250 µm) were cut from the brains of 25–30-day-old Sprague-Dawley rats and incubated for 1 h in normal aCSF saturated with 95% O_2_/5% CO_2_ (pH 7.4). Recordings were made in aCSF containing 2 mM CaCl_2_ and 2 mM MgCl_2_ at 33–35℃. AMPA-receptor (AMPAR)-mediated currents were isolated by adding CGP-55845 (1 µM), D-APV (50 µM), strychnine (1 µM), picrotoxin (100 µM) and S-MCPG (200 µM). The pipette solution contained 117.5 mM Cs-gluconate, 17.5 mM CsCl, 10 mM KOH-HEPES, 10 mM BAPTA, 8 mM NaCl, 5 mM QX-314, 2 mM Mg-ATP, 0.3 mM GTP (pH 7.2, 295 mOsm, resistance 7–9 MΩ). Damaged cells from the slice surface were removed by applying flow of pressurized perfusion solution delivered via a glass pipette (diameter 7–10 µm). Whole-cell voltage-clamp recordings were taken from CA1 pyramidal neurons. Signals were digitized at 10 kHz. Stimulation was applied using a bipolar tungsten electrode placed in the Schaffer collaterals area. Electrical stimuli were delivered every 15 s for 10 min in each experimental episode (application of drugs, change of extracellular [Ca^2+^]); 10 min intervals without stimulation were given between experimental episodes. Miniature (∼20 µm tip diameter) carbon fiber lactate biosensors were placed in the vicinity (within 15–20 µm) of the recorded neurons. Readings of the biosensor currents were taken every 0.5 s, and were synchronized with the recordings of the whole-cell AMPAR-mediated EPSCs.

### Drugs

Carbenoxolone (CBX) in 10 µM concentration and probenecid (150 µM) were used to inhibit pannexin hemichannel activity. CBX (100-200 µM), 5-nitro-2 -(3-phenylpropylamino)-benzoic acid (NPPB) (200 µM) and La^3+^ (100 µM) were used to interfere with connexin hemichannels. MCTs were inhibited with 4-CIN (250–500 µM). Ruthenium red (20 µM) was used to block CALHM1 channels. Tetrodotoxin (1 µM) and muscimol (100 µM) were applied to inhibit neuronal activity. EGTA (1 mM) was used to chelate Ca^2+^. All drugs were from Sigma (Poole, UK).

### Data analysis

Lactate biosensor measurements were processed using 1401 interface and analyzed using *Spike 2* software (Cambridge Electronic Design). Data are reported as mean ± S.E.M. Datasets were compared using paired samples Wilcoxon Signed Ranks Test or Student’s paired or unpaired *t* test, as appropriate. Differences between groups with *p* values of less than 0.05 were considered significant.

## Results

Enzymatic amperometric lactate biosensors were used for real-time recordings of tonic-, hypoxia- and enhanced neuronal activity-stimulated release of lactate in acute slices of rat brainstem, hypothalamus, hippocampus and cortex.

### Hemichannel-mediated tonic release of lactate

Tonic lactate efflux (lactate tone; Figure 2(c)) was detected when lactate biosensors were placed in a direct contact with the uncut ventral surface of the brainstem horizontal slices (278 ± 12 µM; n = 100, *p* < 0.001), coronal hypothalamic slices (349 ± 46 µM; n = 8, *p* = 0.007), or coronal cortical slices (229 ± 15 µM; n = 10, *p* = 0.003). Lactate tone detected on the slice surface was dramatically reduced by application of lactate dehydrogenase inhibitor oxamate (20 mM) (reduction in tone by −76 ± 7%, n = 19, *p* < 0.001, Figure 2(b)), confirming the analyte selectivity of the biosensor detection system.

Opening probability of hemichannels increases when extracellular [Ca^2+^] decreases.^23^ Lowering extracellular [Ca^2+^] (from 2 mM to 0 mM with an addition of 1 mM EGTA to chelate residual Ca^2+^) facilitated release of lactate by the brainstem (by 94 ± 15 µM; n = 22, *p* = 0.001) and cortical (by 109 ± 15 µM; n = 5, *p* = 0.03) slices (Figure 2(c) and (e)). Opening probability of certain connexin hemichannels (e.g. connexin26) also increases in response to CO_2._^15,24,25^ Isohydric hypercapnia (application of aCSF containing 80 mM HCO_3_^−^, *P*CO_2_ 70-80 mmHg, pH 7.4) facilitated lactate release from the ventral surface of the brainstem (by 37 ± 7 µM; n = 10, *p* = 0.003, Figure 2(e)). These data suggested that hemichannels may act as a conduit for lactate release.

To further investigate the role of pannexin/connexin hemichannels in mediating tonic release of lactate, we next applied compounds known to inhibit hemichannel activity. CBX and NPPB which block both pannexin and connexin hemichannels significantly reduced lactate tone (Figure 2(d) and (e)). CBX (100 µM) decreased lactate tone by 65 ± 12 µM (n = 14, *p* < 0.001) in the brainstem slices and by 71 ± 7 µM (n = 5, *p* = 0.03) in the cortical slices. NPPB (200 µM) had a similar effect in the brainstem slices (decrease in lactate tone by 108 ± 17 µM (35%); n = 8, *p* = 0.007). The amount of lactate released in response to lowering extracellular [Ca^2+^] was also markedly reduced in the presence of CBX (−64 ± 22%, n = 8, *p* = 0.02; Figure 2(e)).

Lactate tone was not affected by La^3+^ (100 µM, Figure 2(e)) which is believed to inhibit connexin, but not pannexin hemichannels,^26,27^ suggesting that the latter might be involved. Indeed, inhibition of pannexins by application of CBX at a low concentration (10 µM) or probenecid (150 µM) decreased lactate tone (CBX 10 µM: −29 ± 9 µM, n = 12, *p* = 0.002; probenecid: −31 ± 9 µM, n = 13, *p* = 0.003) (Figure 2(e)). Interestingly, inhibitor of MCTs 4-CIN (250-500 µM) had no effect on tonic release of lactate by either brainstem or cortical slices (Figure 2(d) and (e)). Inhibition of neuronal activity by co-application of tetrodotoxin (TTX, 1 µM) and muscimol (100 µM) was associated with a small reduction in lactate tone (−12 ± 4 µM; n = 9, *p* = 0.01, Figure 2(d) and (e)). Application of ruthenium red (20 µM) to block CALHM1 channels (which are also sensitive to low extracellular [Ca^2+^]^28^) did not reduce the lactate tone (increase by 4.4 ± 1.6 µM, n = 10, Figure 2(e)).

Control tests were performed to determine whether the pharmacological agents used in this study have an effect on lactate biosensor detection system (Figure 1(b)). Only NPPB (200 µM) was found to reduce biosensor current by ∼10%, which is significantly less compared to the effect of the drug on currents generated by the biosensors when positioned on the brain slice (reduction in lactate tone by 35% and reduction in hypoxia-induced lactate release by >50%, see below).

### Connexin hemichannels and MCTs mediate facilitated release of lactate during hypoxia

**Figure 4.**
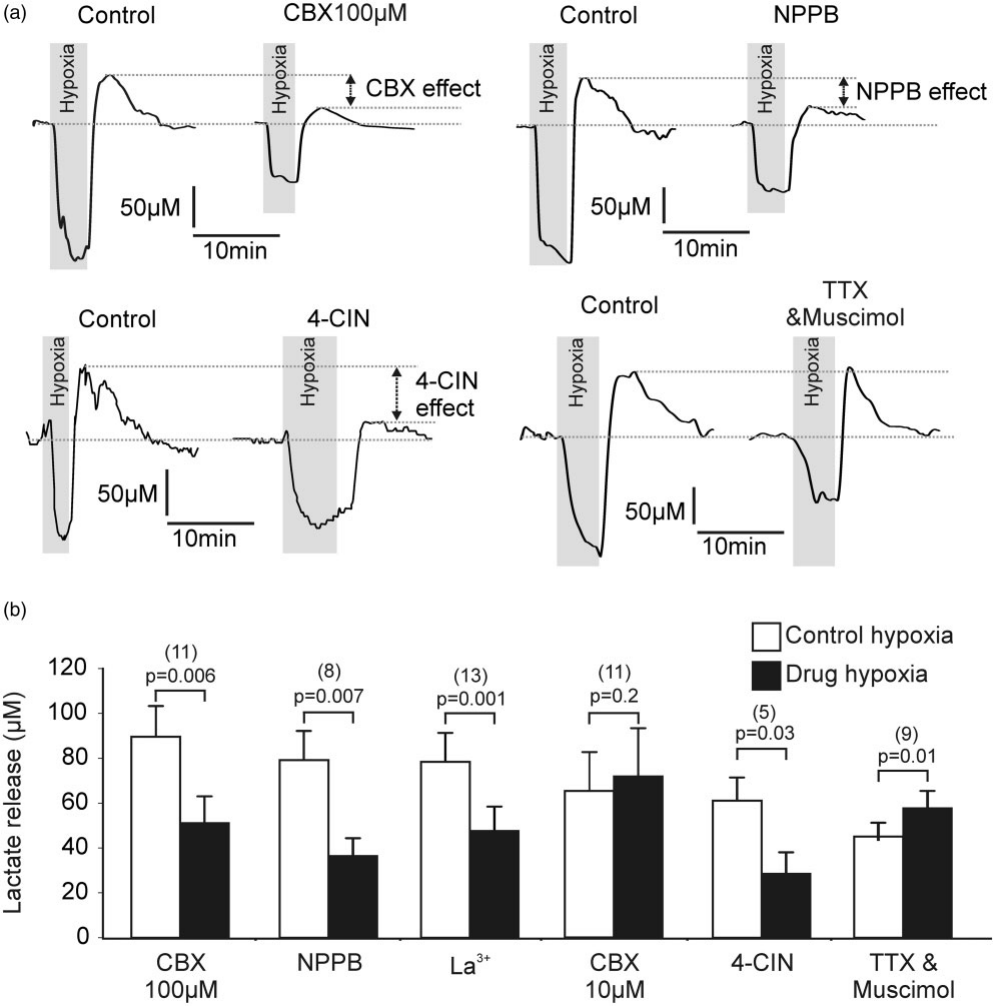
Connexin hemichannels and monocarboxylate transporters (MCTs) mediate facilitated release of lactate during hypoxia. (a) Representative recordings of changes in lactate biosensor current showing hypoxia-induced lactate release by the brainstem slices in the absence and presence of CBX (100 µM), NPPB (200 µM), 4-CIN (250 µM) or co-application of TTX (1 µM) and muscimol (100 µM); (b) Summary data illustrating peak hypoxia-induced lactate release in the absence and presence of CBX, NPPB, La^3+^, 4-CIN, TTX+muscimol.

Tissue hypoxia decreases mitochondrial utilization of pyruvate leading to an increase in lactate production and release. Since biosensors require oxygen to operate (*lactate oxidase* entrapped in the biosensor polymer layer converts lactate to pyruvate and H_2_O_2_ which is detected electrochemically), decreases in O_2_ concentration (saturation of the media with 95%N_2_/5%CO_2_) reduce biosensor currents (Figure 3(a) and (b)). Therefore, lactate release induced by hypoxia can only be determined upon re-oxygenation^22^ (increase in lactate release in the brainstem slices from 219 ± 16 µM to 284 ± 18 µM; n = 61; *p* < 0.001; in the cortical slices from 182 ± 10 µM to 302 ± 26 µM; n = 5; *p* = 0.03). These values represent an accurate estimation of peak hypoxia-induced lactate release since lactate efflux of a similar magnitude is triggered by bath application of the mitochondrial complex III inhibitor myxothiazol (Figure 3(b) and (c)). Hypoxia-induced lactate release was not affected by CBX applied at a low concentration (10 µM) (Figure 4(b)) or probenecid, suggesting that pannexins are not involved. However, lactate release triggered by hypoxia was significantly (by ∼50%) reduced in the presence of either MCTs inhibitor 4-CIN (250 µM, *p* = 0.03) or any of the tested connexin hemichannel blockers (CBX 100 µM, *p* = 0.006; NPPB, *p* = 0.007; or La^3+^, *p* = 0.001) (Figure 4(a) and (b)). Blockade of the neuronal activity with TTX and muscimol resulted in a slightly higher peak lactate release in response to hypoxic challenge (Figure 4(a) and (b)).

### Connexin hemichannels contribute to the release of lactate triggered by enhanced neuronal activity

**Figure 5.**
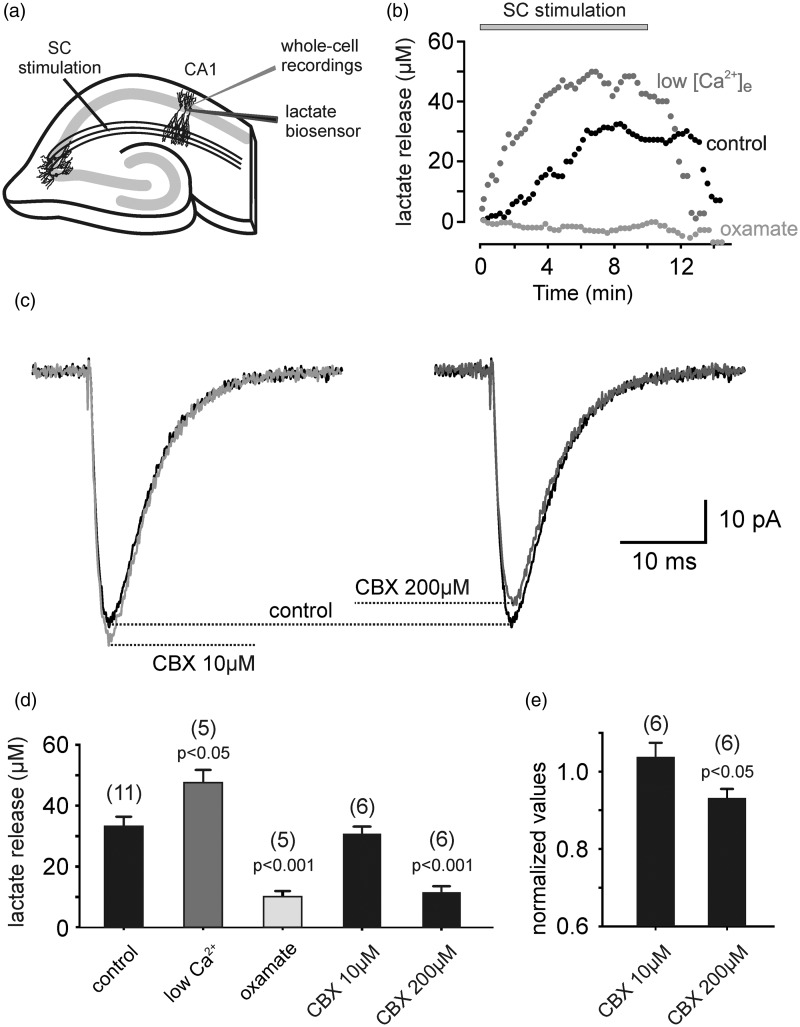
Connexin hemichannels contribute to the increases in lactate release triggered by enhanced neuronal activity. (a) Schematic drawing of the experimental design. Miniature (20 µm) carbon fiber lactate biosensors were placed in the vicinity of the patch-clamped hippocampal CA1 neurons to record lactate release in response to electrical stimulation of Schaffer collateral (SC) fibers; (b) Representative recordings of changes in biosensor current illustrating lactate release in CA1 in response to stimulation of Schaffer collaterals in control and low (0.2 mM) extracellular [Ca^2+^] conditions and in the presence of oxamate (20 mM). Schaffer collaterals were stimulated for 10 min; (c) Representative AMPAR EPSCs recorded in the same experiment in the absence and presence of CBX (each trace is an average of 10); (d) Summary data illustrating peak lactate release in CA1 area in response to stimulation of Schaffer collaterals in control and low extracellular [Ca^2+^] conditions, in the presence of oxamate or CBX; (e) Summary data illustrating amplitudes of AMPAR-mediated EPSCs normalized to the control values.

We next examined hemichannel involvement in the release of lactate driven by enhanced neuronal activity.^29^ Miniature (∼20 µm in diameter) carbon fiber lactate biosensors placed in the vicinity of the patch-clamped hippocampal CA1 neurons (Figure 5(a)) recorded significant (peak release 35 ± 3 µM, n = 11) lactate release in response to stimulation of Schaffer collateral fibers (Figure 5(b) and (d)). Lactate release in CA1 in response to stimulation of Schaffer collaterals was increased in low extracellular [Ca^2+^] (0.2 mM) conditions (peak release 48 ± 4 µM, n = 5, *p* < 0.05; Figure 5(b) and (d)), abolished in the presence of oxamate applied 20 min prior (n = 5, *p* < 0.001; Figure 5(b) and (d)), and markedly reduced by CBX (peak release 12 ± 2 µM, n = 6; *p* < 0.001; Figure 5(d)). Simultaneous recordings of AMPA-receptor-mediated EPSCs showed that decreased release of lactate in the presence of CBX is associated with small decreases in the amplitude of the evoked EPSCs (Figure 5(c) and (e)). CBX applied at a low concentration (10 µM) had no effect on the evoked EPSCs and activity-dependent lactate release (Figure 5(c) to (e)).

## Discussion

Lactate is continuously produced by the brain parenchyma even in conditions of ample oxygen supply.^30^ Glycolysis in the presence of oxygen (aerobic glycolysis) is an interesting feature of brain metabolism, and in resting conditions there is a net efflux of lactate from the CNS, i.e. lactate concentration in the venous blood is higher than in the arterial blood supplying the brain.^31,32^ For decades it has been thought that MCTs are the only pathway of lactate transport from the cells which produce it. This dogma was challenged by the results of the recent study suggesting that lactate release may occur via the mechanisms involving certain membrane channels.^11^ The data obtained in the present study suggest that these alternative mechanisms include pannexin and functional connexin hemichannels. We report that hemichannels are capable to function as a conduit of lactate release and this mechanism is recruited during hypoxia and periods of enhanced neuronal activity.

We show that tonic efflux of lactate can indeed be detected in hyperoxygenated environment (*P*O_2_ > 500 mmHg in standard conditions used for the experiments in brain slices superfused with aCSF saturated with 95% O_2_/5% CO_2_) in reduced preparations, such as acute brainstem, hypothalamic or cortical slices. Lactate tone detected on the intact ventral brainstem surface was similar to that recorded by the biosensors placed in a direct contact with coronal brain sections, indicating that damaged cells are not the primary source of this release. Lactate tone increased in conditions of low extracellular [Ca^2+^] or high *P*CO_2_, which favor hemichannel opening,^15,24,25,33^ and decreased in the presence of pharmacological agents known to inhibit pannexin and connexin hemichannels. Efflux of lactate facilitated by hypoxia was significantly reduced by either connexin or MCT blockers. Lactate release in CA1 triggered in response to stimulation of Schaffer collaterals was enhanced in low extracellular [Ca^2+^] and reduced by CBX. This is interesting because lowering extracellular [Ca^2+^] should reduce the synaptic strength and affect neuronal responses in the projection area. This observation suggests that release of lactate might be uncoupled from synaptic activity responding to ionic movements associated with axonal and dendritic membrane potentials propagation. For example, there is recent evidence showing that an increase in extracellular concentration of K^+^ (within the physiological range) is sufficient to trigger release of lactate by astrocytes.^11^ Through hemichannels or other unidentified channels,^11^ the lactate anion (Lac^−^) can exit cells without the involvement of the proton and facilitate Na^+^ entry (through the activity of sodium-coupled MCTs), which is required by the Na^+^/K^+^ATPase for uptake of neuronally released K^+^.

It needs to be acknowledged that none of the pharmacological compounds available today are specific hemichannel blockers. Nevertheless, they all have different off target effects strongly suggesting that hemichannels are responsible for this lactate release. Facilitated lactate release in response to lowering extracellular [Ca^2+^] or increases in tissue *P*CO_2_ further supports this conclusion. Our pharmacological survey also suggests that other membrane channels sensitive to changes in extracellular [Ca^2+^] (e.g. CALHM1, effectively blocked by Ruthenium red) are unlikely to be involved.

These results provide strong evidence that MCTs are not the sole conduit of lactate release in the CNS. It is rather surprising that the evidence of membrane channel-mediated lactate release was only recently reported,^11^ considering that molecules as large as ATP and glucose are known to be transported via hemichannel opening.^12–18,25^ All cells of the brain parenchyma (neurons, astrocytes, oligodendrocytes and microglia) express pannexins and connexins and identification of the exact source(s) of lactate release detected by the biosensors on the surface of the brain slice *in vitro* is beyond the scope of the present study. Nevertheless, the existing evidence points to astroglia as an important source of lactate release. Recent studies involving measurements of cytosolic lactate concentration in astrocytes using genetically encoded fluorescence sensors reported values of ∼1.4 mM and concluded that at resting conditions astrocytes maintain a steady-state reservoir of lactate allowing its rapid mobilization.^11^ Pharmacological compounds which are expected to significantly reduce neuronal activity (TTX and muscimol) had only a small effect on tonic lactate release, while astroglial activity appears to correlate well with lactate production and release by these cells.^2,6,11^

In summary, we show that pannexin and/or connexin hemichannels are capable of functioning as a conduit of lactate transport across the membrane and this mechanism is recruited in conditions of low *P*O_2_, high *P*CO_2_ or during periods of enhanced neuronal activity. The identified mechanism may play a role in providing rapid and effective local supply of lactate to meet increased metabolic demands of active neurons. Indeed, extracellular [Ca^2+^] can decrease to ∼0.7 mM during periods of intense neuronal activity.^34–37^ This would increase opening probability of hemichannels expressed by neighbouring astrocytes leading to highly localized neuronal activity-driven release of lactate “on-site”. Neuronal activity-dependent opening of connexin hemichannels triggered by decreases in extracellular [Ca^2+^] (and leading to the release of ATP) has been demonstrated previously.^38^ At rest, hemichannel-mediated mechanisms may contribute to the convective flux of lactate across the astrocytic syncytium and its clearance from the brain via the circulation^3^ and/or the glymphatic system.^39^ During physical activity, when peripheral lactate is fuelling our brains, hemichannels are hypothesized to play an important role in the effective uptake of this metabolic substrate from the arterial blood.
